# The Genome Characteristics and Predicted Function of Methyl-Group Oxidation Pathway in the Obligate Aceticlastic Methanogens, *Methanosaeta* spp

**DOI:** 10.1371/journal.pone.0036756

**Published:** 2012-05-10

**Authors:** Jinxing Zhu, Huajun Zheng, Guomin Ai, Guishan Zhang, Di Liu, Xiaoli Liu, Xiuzhu Dong

**Affiliations:** 1 State Key Laboratory of Microbial Resources, Institute of Microbiology, Chinese Academy of Sciences, Beijing, People’s Republic of China; 2 Graduate School, Chinese Academy of Sciences, Beijing, People’s Republic of China; 3 Shanghai-MOST Key Laboratory of Health and Disease Genomics, Chinese National Human Genome Center at Shanghai, Shanghai, People’s Republic of China; 4 Information center, Institute of Microbiology, Chinese Academy of Sciences, Beijing, People's Republic of China; University of Groningen, The Netherlands

## Abstract

In this work, we report the complete genome sequence of an obligate aceticlastic methanogen, *Methanosaeta harundinacea* 6Ac. Genome comparison indicated that the three cultured *Methanosaeta* spp., *M. thermophila*, *M. concilii* and *M. harundinacea* 6Ac, each carry an entire suite of genes encoding the proteins involved in the methyl-group oxidation pathway, a pathway whose function is not well documented in the obligately aceticlastic methanogens. Phylogenetic analysis showed that the methyl-group oxidation-involving proteins, Fwd, Mtd, Mch, and Mer from *Methanosaeta* strains cluster with the methylotrophic methanogens, and were not closely related to those from the hydrogenotrophic methanogens. Quantitative PCR detected the expression of all genes for this pathway, albeit ten times lower than the genes for aceticlastic methanogenesis in strain 6Ac. Western blots also revealed the expression of *fwd* and *mch*, genes involved in methyl-group oxidation. Moreover, ^13^C-labeling experiments suggested that the *Methanosaeta* strains might use the pathway as a methyl oxidation shunt during the aceticlastic metabolism. Because the *mch* mutants of *Methanosarcina barkeri* or *M. acetivorans* failed to grow on acetate, we suggest that *Methanosaeta* may use methyl-group oxidation pathway to generate reducing equivalents, possibly for biomass synthesis. An *fpo* operon, which encodes an electron transport complex for the reduction of CoM-CoB heterodisulfide, was found in the three genomes of the *Methanosaeta* strains. However, an incomplete protein complex lacking the FpoF subunit was predicted, as the gene for this protein was absent. Thus, F_420_H_2_ was predicted not to serve as the electron donor. In addition, two gene clusters encoding the two types of heterodisulfide reductase (Hdr), *hdrABC*, and *hdrED*, respectively, were found in the three *Methanosaeta* genomes. Quantitative PCR determined that the expression of *hdrED* was about ten times higher than *hdrABC*, suggesting that *hdrED* plays a major role in aceticlastic methanogenesis.

## Introduction

Methanogenic *Archaea* are the only organisms known to produce abundant CH_4_ for energy metabolism. Therefore, they exert a significant ecological impact on global carbon cycling. Cultured methanogens are categorized into four metabolic types based on methanogenic precursors, including hydrogenotrophic, methylotrophic, aceticlastic and methanol plus H_2_ methanogenesis [1]. Since an estimated two-thirds of the methane in nature is from acetate [2], aceticlastic methanogenesis makes a major contribution to global methane production. So far, the methanogens utilizing acetate for methanogenesis are confined to the order *Methanosarcinales*. The genus *Methanosarcina* (*Msr.*) consists of the most metabolically diverse methanogenic species, most of them conduct three types of methanogenic metabolism [3]. In general, *Methanosarcina* strains have large genomes, e.g. 5.8 Mb for *Msr. acetivorans*, 4.8 Mb for *Msr. barkeri* and 4.1 Mb for *Msr. mazei* [4], [5], [6], and are about two times larger than other sequenced methanogen genomes.

In contrast, species of the other genus of aceticlastic methanogens *Methanosaeta* are obligately aceticlastic. As specialists, *Methanosaeta* strains possess a higher affinity for acetate than *Methanosarcina* stains and are favored in environments with low concentrations of acetate [7]. Hence, they are deemed the principal players in aceticlastic methanogenesis in nature.

Thus far, only three *Methanosaeta* species have been cultured. *M. harundinacea* is a mesophilic species isolated from an upflow anaerobic sludge blanket reactor treating beer-manufacturing wastewater in Beijing [8]. To gain insight into the genetic background of the aceticlastic methanogens, the complete genome of *M. harundinacea* 6Ac was sequenced and compared to that of the two other species. In addition to the other common genome characteristics among the three species, unexpectedly the methyl-group oxidation pathway was present in all three genomes. In methylotrophic methanogens, this pathway provides reducing equivalents for methanogenesis [9]. It also plays a role in the anaerobic oxidation of methane [10], [11]. To understand the possible function of the pathway, in this study, the expression of the genes in the pathway was tested in *M. harundinacea* 6Ac. The gene expression and the preliminary physiological study suggest that the methyl-group oxidation pathway can be used to generate the extra reducing equivalents in the obligate aceticlastic methanogens.

**Figure 1:**
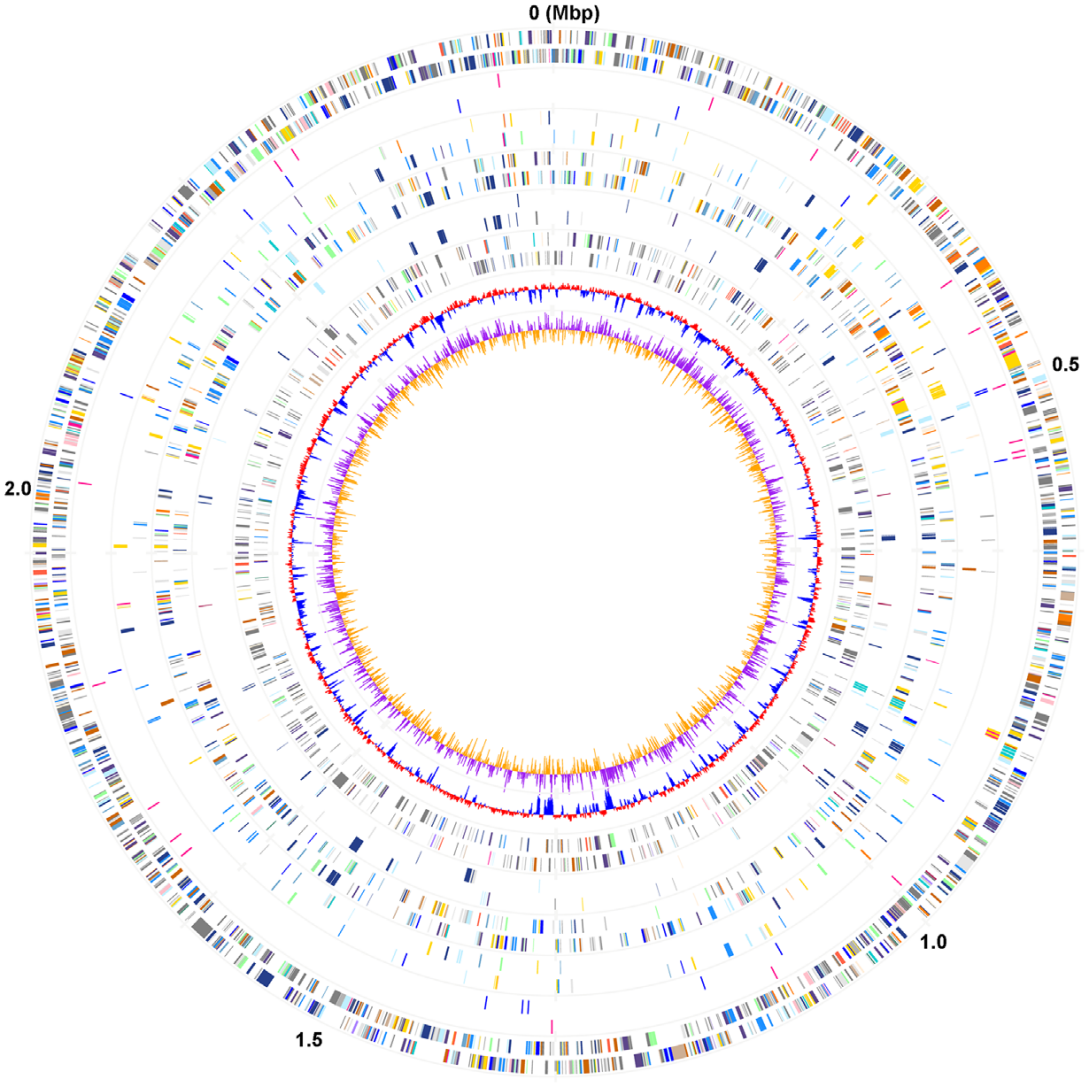
Circular representation of the *M. harundinacea* genome. The circles show (innermost to outermost): (**1**) GC skew; (**2**) G+C content; (**3**) genes included in the genome of *M. harundinacea* but not in the genomes of *M. concilii* and *M. thermophila*; (**4**) genes involved in methane metabolism; (**5**) genes found in all the three *Methanosaeta* genomes; (**6**) core genes found in the methanogen genomes sequenced; (**7**) RNA genes, including tRNA (red) and rRNAs (yellow); (**8**) genes on the plus and minus strand, respectively. All the genes were colored by functional categories according to COG classification.

## Results and Discussion

### General genome features of *Methanosaeta* species

In this study, the complete genome of *Methanosaeta harundinacea* 6Ac, which contains a single circular chromosome and a circular plasmid designated as pH 6AC, was obtained. The chromosome is 2,559,043 bp in length with an average G+C content of 60.6% (Figure 1). It contains one rRNA operon (5S, 16S, and 23S), a distinct 7S rRNA gene, and 39 tRNA genes (Table 1). There are 2,353 coding sequences (CDSs) with an average length of 937 bp in the chromosome, representing 85% of the entire genome. Of the protein-coding genes, 69.7% of the CDSs (1,640) were assigned to the functional categories of Cluster of Orthologous Groups (COG). Approximately 22.4% (528/2,353) of the chromosomal gene products are hypothetical proteins, accounting for the majority of the *M. harundinacea*-specific genes (226/305) when compared with other available *Methanosarcinales* genomes. The plasmid pH 6AC is 11,991 bp in length and carries 18 predicted ORFs, with 16 of these encoding hypothetical proteins.

**Table 1:** General genome features of the aceticlastic methanogens, *Methanosaeta* spp. and *Methanosarcina* spp.

General	*Msa. harundinacea*	*Msa. concilii*	*Msa. thermophila*	*Msr. acetivorans*	*Msr. barkeri*	*Msr. mazei*
GenBank accession no.	CP003117	NC_015416	NC_008553	NC_003552	NC_007355	NC_00391
Size (bp)	2,559,043	3,008,626	1,879,471	5,751,492	4,837,408	4,096,345
G+C content (%)	60.63	51	53.5	42.7	39.2	41.5
Protein-coding genes	2,353	2,906	1,781	4,540	3,698	3,371
Coding Regions (%)	81	83	82	74	70	75
rRNA operons	1	2	2	3	3	3
tRNA genes	39	44	48	59	62	57
Plasmid	1	1	0	1	1	0

Abbreviations: *Msa.*, *Methanosaeta*; *Msr.*, *Methanosarcina*.

**Table 2:** Gene numbers in each functional category present in the three genomes of *Methanosaeta*.

Category of Function	*M. harundinacea*	*M. concilii*	*M. thermophila*
Amino acid transport and metabolism	137	148	107
Carbohydrate transport and metabolism	55	58	47
Cell division and chromosome partitioning	16	19	10
Cell envelope biogenesis, outer membrane	58	85	57
Cell motility and secretion	11	14	5
Coenzyme metabolism	127	130	106
Defense mechanisms*	30	32	15
DNA replication, recombination, and repair	104	266	97
Energy production and conversion	165	164	122
General function prediction only	291	318	191
Inorganic ion transport and metabolism	136	160	94
Intracellular trafficking and secretion	16	25	14
Lipid metabolism	24	29	22
Nucleotide transport and metabolism	59	53	48
Posttranslational modification	93	100	58
Secondary metabolites biosynthesis	16	19	11
Signal transduction mechanisms	37	33	30
Transcription	85	104	65
Translation, ribosomal structure and biogenesis	157	152	145
Unknown Function	274	307	188
No homolog	439	592	244

* , Proteins in this category could enable an organism to resist exogenous compounds and genetic materials, thus prevent environment damages and maintain the genetic stability [33], [45].

A genome sequence comparison analysis for the three *Methanosaeta* strains, *M. concilii* GP6, *M. thermophila* PT, and *M. harundiacea* 6Ac, did not reveal an obvious colinearity. However, a remarkable difference among the three *Methanosaeta* species was observed in terms of the genome sizes, resulting in the difference in gene content (Table 1), with *M. thermophila* PT having the smallest genome, and *M. concilii* having more genes in category of DNA replication, recombination and repair, which is contributed by the multiple copies of the genes in this category. The coding regions in the three genomes of the *Methanosaeta* strains (>80%) account for the higher percentage of total genes compared to those of the *Methanosarcina* strains (70–75%).

Comparison of the gene function categories among the three species (Table 2) showed that while the two mesophilic strains contain similar gene numbers in each functional category, the thermophilic one has remarkably reduced gene numbers in cell motility and secretion, defense systems, and post-translational modification (PTM). This is probably consistent with its hot niche, where the biodiversity is relatively lower.

### A suite of genes for the methyl-group oxidation pathway present in the *Methanosaeta* genomes

**Figure 2:**
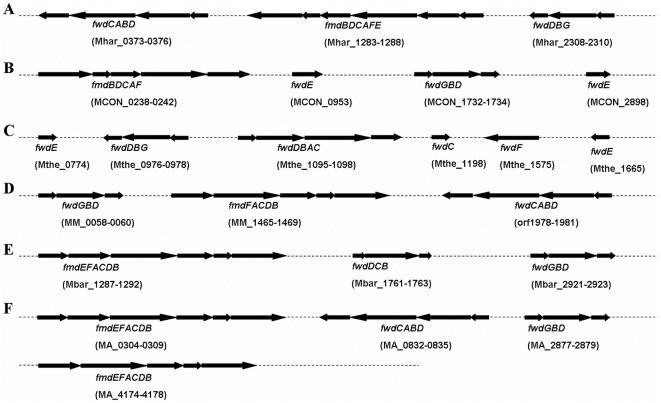
Gene organizations of formylmethanofuran dehydrogenase (*fmd* or *fwd*) in the genomes of *M. harundinacea* (A), *M. concilii* (B), and *M. theromphila* (C) compared with those in the genomes of *Msr. mazei* (D), *Msr. barkeri* (E) and *Msr. acetivorans* (F).

It has been long determined that *Methanosaeta* strains are obligately aceticlastic methanogens, acetate being the exclusive substrate for CH_4_ formation and energy biosynthesis. However, an entire suite of genes for methyl-group oxidation pathway was found in the three genomes. Two types of formylmethanofuran dehydrogenase, tungsten formylmethanofuran dehydrogenase (Fwd) and the molybdenum isoenzyme (Fmd), were found. As shown in Figure 2, *M. thermophila* possesses only the tungsten formylmethanofuran dehydrogenase genes, implying that the tungsten formylmethanofuran dehydrogenase is essential for these methanogens. This assumption could be supported by the observation of Hochheimer *et al*. [12] that the tungsten enzymes are constitutively expressed, while the molybdenum ones show a molybdate-induced expression in two thermophilic methanogens *Methanothermobacter wolfeii* and *Methanothermobacter thermoautotrophicus*.

**Figure 3:**
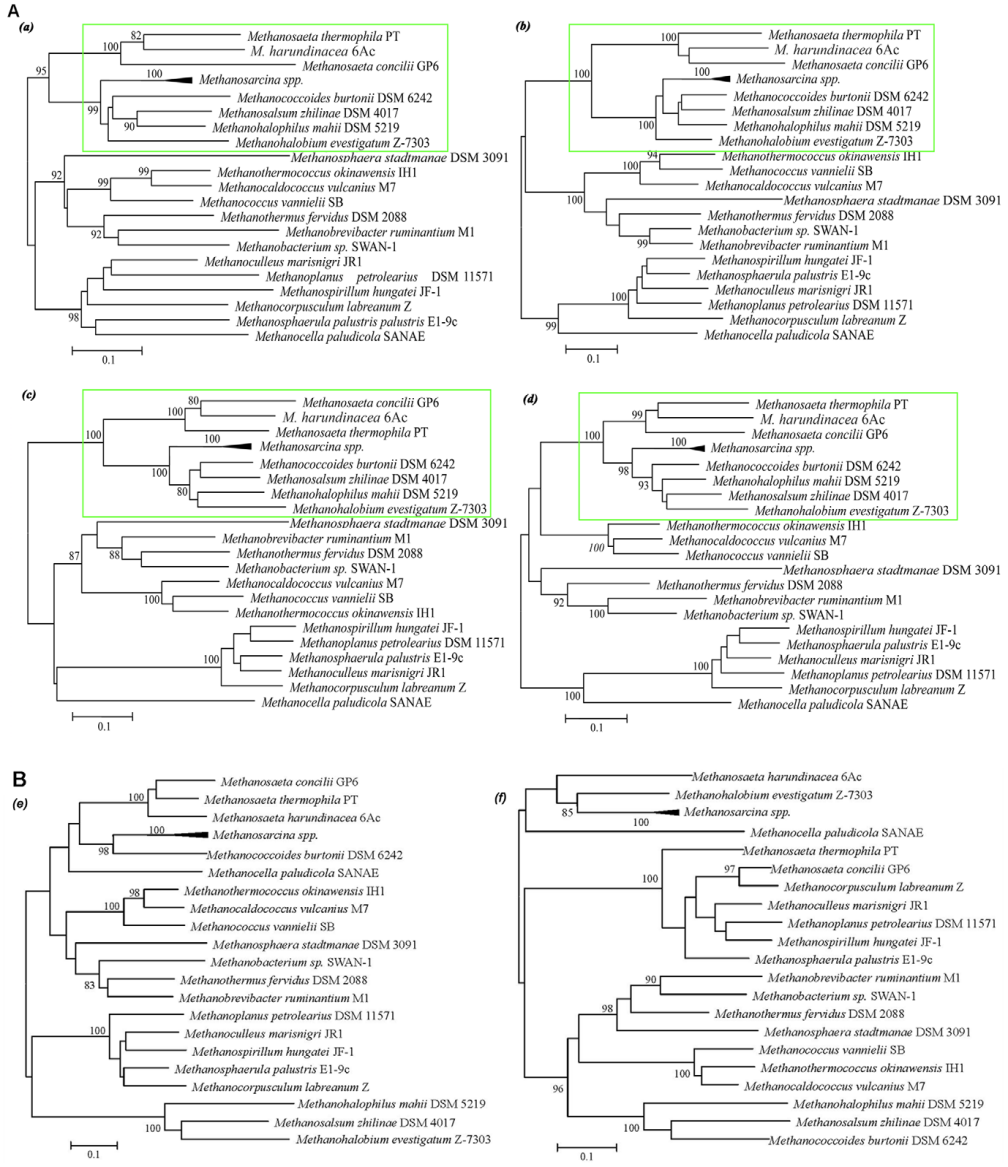
Phylogeny of enzymes from the pathway of methanogenesis. A. representative enzymes from the C-1 branch. B. other representative enzymes. a. Formylmethanofuran-tetrahydromethanopterin formyltransferase (Ftr), b. N^5^N^10^-methenyl-tetrahydromethanopterin cyclohydrolase (Mch), c. F_420_-dependent N^5^N^10^-methylene-tetrahydromethanopterin dehydrogenase (Mtd), d. and F_420_-dependent N^5^N^10^-methylene- tetrahydromethanopterin reductase (Mer), e. methyltetrahydrosarcinapterin: CoM methyltransferase A (MtrA), f. MtrH. Protein sequences were aligned with ClustalX [43] and phylogenetic analysis was performed with MEGA [44] using the neighbor-joining algorithm. Bootstrap support was obtained from neighbor-joining (first value), maximum-parsimony (second value) and maximum-likelihood (third value) methods based on 1000 replicates. The accession number of each reference sequence is shown in table S2. Framed sequences refer to those involved in the methyl methanogenesis pathway. The bar represents 10% estimated sequence divergence.

**Figure 4:**
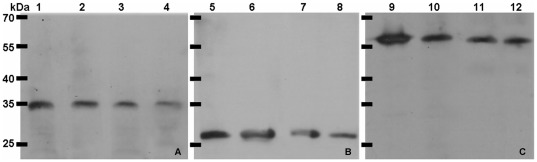
Western blot assays for FwdC (A), Mch (B) and McrA (C). Lane 1, 5 and 9, 0.2 µg purified FwdC, Mch and McrA as the positive control; lane 2, 6 and 10, 10 µg cell extract from the pre-exponential phase culture; lane 3, 7 and 11, 10 µg cell extract from the exponential phase culture; lane 4, 8 and 12, 5 µg cell extract from the stationary phase culture. The molecular masses of subunit FwdC, Mch and McrA are about 27.5 kDa, 34 kDa, and 61 kDa, respectively. The migrations of molecular mass standards are shown on the left of pictures.

**Table 3:** Transcript abundance of the genes for methyl-group oxidation pathway in *M. harundinacea* 6Ac estimated by QPCR.

Gene	Gene product	Relative Expression*
Mhar_0751	Acs	15.6±1.7
Mhar_0789	CdhA	14.6±1.3
Mhar_2323	CdhD	12.4±1.2
Mhar_0495	McrB	131±9
Mhar_0498	McrA	128±11
Mhar_2090	MtrE	15.6±2.2
Mhar_2091	MtrD	14.3±1.6
Mhar_2092	MtrC	15.7±1.8
Mhar_2093	MtrB	14.9±1.6
Mhar_2094	MtrA	14±1.2
Mhar_2095	MtrF	13.5±1.1
Mhar_2096	MtrG	14.8±1.4
Mhar_2097	MtrH	16±1.6
Mhar_0792	HdrE	7±0.6
Mhar_0793	HdrD	13±0.7
Mhar_0604	HdrB	2.1±0.3
Mhar_0605	HdrC	1.7±0.2
Mhar_0607	HdrA	2.5±0.3
Mhar_0373	FwdC	3.6±0.6
Mhar_0374	FwdA	2.8±0.5
Mhar_0375	FwdB	0.8±0.1
Mhar_0376	FwdD	0.7±0.1
Mhar_1283	FwdB	2.1±0.3
Mhar_1284	FwdD	2.4±0.3
Mhar_1285	FwdC	0.8±0.1
Mhar_1286	FwdA	4.3±0.7
Mhar_1287	FwdF	3.4±0.5
Mhar_1288	FwdE	1.1±0.2
Mhar_1285	FwdC	0.8±0.1
Mhar_1286	FwdA	4.3±0.7
Mhar_1287	FwdF	3.4±0.5
Mhar_1288	FwdE	1.1±0.2
Mhar_2214	Ftr	1.4±0.2
Mhar_2174	Mch	1.7±0.3
Mhar_1470	Mtd	1.0±0.2
Mhar_0856	Mer	3.4±0.6

* , copy number of gene/copies of 16S rRNA gene×10^5^.

**Table 4:** Percentage of ^13^C-labeled acetate, ^13^C-labeled CO_2_ and ^13^C-labeled CH_4_ formation in the 60-day cultures in a medium with 5% ^13^C-labeled acetate, NaHCO_3_ or CH_4_.

Labeled substances added	acetate	CO_2_	CH_4_
	[^13^C/(^12^C+^13^C), (%)]		
Control	1.0706±0.0004	1.0779±0.0008	1.0648±0.0006
^13^CH_4_	1.0713±0.0006	1.0774±0.0007	4.6393±1.5682
^13^CH_3_COO_2_^−^	5.0329±0.2512	1.1072±0.0203	5.1261±0.5427
CH_3_^13^COO_2_^−^	4.7927±0.3382	4.2723±0.7236	1.0661±0.0005
H^13^CO_3_^−^	1.0715±0.0007	4.0664±0.8178	1.0706±0.0007

**Figure 5:**
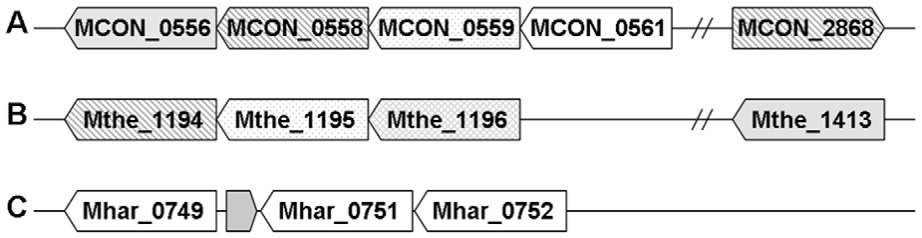
Organization of *acs* genes in *M. concilii* (A), *M. thermophila* (B), and *M. harundinacea* (C). Closest homologs between species are indicated by the identical patterns.

Like the genes in hydrogenotrophic methanogens, only one gene copy is present for each of formylmethanofuran-tetrahydromethanopterin formyltransferase (Ftr), N^5^N^10^-methenyl-tetrahydromethanopterin cyclohydrolase (Mch), F_420_-dependent N^5^N^10^-methylene-tetrahydromethanopterin dehydrogenase (Mtd), and F_420_-dependent N^5^N^10^-methylene- tetrahydromethanopterin reductase (Mer).

To gain an insight into the gene organization for methyl-group oxidation pathways in the *Methanosaeta* genome, we found that genes for the formylmethanofuran dehydrogenase complex were arranged differently in the three strains. While the three operons for the Fwd complex in *M. harundinacea* 6Ac are arrayed in a similar pattern to those of *Methanosarcina* spp., only two *fmd*/*fwd* operons were present in *M. concilii* and *M. thermophila*, with additional *fwd*E genes scattered on the chromosomes (Figure 2).

Based on the protein phylogenetic analysis, we found that genes encoding Fwd, Mtd, Mch, and Mer, from the three *Methanoseata* strains were closely related with those from the methylotrophic methanogens, which branched off from those of the hydrogenotrophic methanogens (Figure 3A). However, other proteins that functioned in different methanogenic pathways did not form similar clades (Figure 3B). The different phylogenetic clustering patterns for the proteins can be attributed to their varied roles, such as Fwd, Mtd, Mch, and Mer in the methylotrophic methanogens mainly act in methyl oxidation, while in the hydrogenotrophic methanogens they are involved in CO_2_ reduction to methane formation.

### Genes encoding the proteins involved in methyl-group oxidation pathway expressed but at relatively low levels

To examine whether the genes for methyl-group oxidation pathway were expressed during growth, transcription of the genes in *M. harundinacea* 6Ac was detected by means of quantitative PCR. The assay showed that the transcript abundances were at an average level of about 14% equivalence to that of the *N^5^*-methyl-H_4_SPT: CoM methyltransferase *(mtr)* genes (Table 3), but were 1.58 times above the average levels of the total genes based on microarray data (unpublished data). Furthermore, Western blot also determined the expression of the C subunit of Fwd and Mch in the acetate-growing culture of *M. harundinacea* 6Ac (Figure 4). Therefore, an active methyl-group oxidation pathway was present in strain 6Ac even during aceticlastic growth.

### Methyl-group oxidation pathway could act as a methyl oxidation shunt of acetate in *Methanosaeta* strains

To reveal the possible function of the methyl-group oxidation pathway in the obligate aceticlastic methanogens, ^13^CH_4_ or NaH^13^CO_3_ was added to cultures of strain 6Ac to determine if trace methane oxidation or production occurred. However, neither the ^13^CO_2_ in the ^13^CH_4_ supplemented culture nor the ^13^CH_4_ in the NaH^13^CO_3_ supplemented culture was detected after 60 days of incubation. These tests indicated that the methyl-group oxidation pathway in *M. hurandiacea* 6Ac was not used for trace methane oxidation or production. Cultures were then supplemented with 1-^13^C-labeled acetate or 2-^13^C-labeled acetate. Upon acetate depletion by incubation at 37°C for 60 days, as expected in the [1-^13^C] acetate-culture only labeled ^13^CO_2_ but not ^13^CH_4_ was found. Surprisingly, in the [2-^13^C] acetate-culture, ^13^CH_4_ and ^13^CO_2_ were both detected. The generated ^13^CO_2_ accounted for about 1% of the methyl carbon of acetate (Table 4). This suggested that 1% methyl carbon of acetate was oxidized to CO_2_ through methyl-group oxidation pathway in the obligate aceticlastic *Methanosaeta* strains. This oxidative carbon flux shunt, like in methylotrophic methanogenesis, was probably used by *Methanosaeta* strains to generate essential reducing equivalents such as reduced ferredoxin or reduced F_420_ for other biological processes, e.g. cell biomass synthesis. This hypothesis is supported by the fact that *Msr. barkeri* and *Msr. acetivorans* fail to grow on acetate upon inactivation of *mch*, which is required for methyl group oxidation [13].

### Characteristics of the genes involved in aceticlastic methanogenesis pathways in *Methanosaeta* strains

Acetate in undissociated form (pKa = 4.75, 25°C) is believed to diffuse freely across the cytoplasmic membrane of bacteria [14]. However, under neutral pH, in which *Methanosaeta* strains grow optimally, acetate is present in a dissociated state, thus, a transport protein is necessary. A putative acetate transporter, the Ady2 gene (Mhar_0433), was present in the three *Methanosaeta* genomes and has been identified as an active acetate transporter in *Saccharomyces cerevisiae* based on microarray analyses of an *ady2*Δ strain [15].

It is believed that the first reaction, i.e. acetate activation to acetyl-CoA, is a rate-limiting reaction for aceticlastic methanogenesis [16], [17]. Acetate activation is accomplished through different reactions in the two types of aceticlastic methanogens, i.e. *Methanosaeta* strains use AMP-forming acetyl-CoA synthetase (ACS), while *Methanosarcina* spp. employ the combined actions of acetate kinase (AK) and phosphotransacetylase (PTA). Surprisingly, three to five *acs* gene homologs were identified in the genomes of *Methanosaeta* spp., and most of them were organized in tandem (Figure 5). Quantitative PCR assay showed that the three *acs* genes in *M. harundinacea* 6Ac were expressed differentially. The abundance of *acs2* (Mhar_0751) and *acs3* (Mhar_0752) transcripts were about nine fold higher than those of *acs1* (Mhar_0749) (Table 3). This suggests that *acs2* and *acs3* might be the key proteins for aceticlastic methanogenesis, while *acs1* may be more highly expressed under growth conditions not examined here. As biochemically characterized, following the acetate activation step, *Methanosarcina* and *Methanosaeta* employ the same enzymes in the remaining reactions in aceticlastic methanogens. The corresponding genes encoding the proteins involved in those reactions are all present in the three *Methanosaeta* genomes; these include the genes for the carbon monoxide dehydrogenase/acetyl-CoA decarbonylase complex (CODH/ACDS), methyltetrahydrosarcinapterin: CoM methyltransferase (Mtr), methyl-CoM methylreductase (Mcr), and heterodisulfide reductase (Hdr).

### Differential expression of two operons encoding two types of heterodisulfide reductase

As in the *Methanosarcinales* genomes, two gene classes of coenzyme B-coenzyme M heterodisulfide reductase, *hdrABC* and *hdrED*, are present in the genomes of *Methanosaeta* strains. Quantitative PCR assay showed that the transcript abundances of *hdrED* are about ten times higher than those of *hdrABC* in strain 6AC (Table 3), indicating that *hdrED* is the primary Hdr in methanogenesis in *M. harundinacea* 6AC. According to that HdrA1B1C1 is used specifically in the methylotrophic methanogenesis pathway in *Methanosarcina acetivorans* [18], we hypothesize that *hdrED* and *hdrABC* obtain reducing equivalents from CODH and the methyl-group oxidation in *Methanosaeta* strains, respectively; and those obtain from methyl-group oxidation by *hdrABC* can be used to compensate the biosynthesis-consumed reducing equivalents.

### An *fpo* operon for a truncate electron transport complex possibly functions in the reduction of CoM-CoB heterodisulfide

In *Methanosarcina* species, a heterodisulfide reductase is involved in releasing coenzyme M and coenzyme B from the heterodisulfide using reduced ferredoxin as electron donor as well as energy conservation in acetate metabolism [19]. To implement this biochemical reaction, *Msr. mazei* and *Msr. barkeri* employ the Ech complex and F_420_ non-reducing hydrogenase, while *Msr. acetivorans* uses the Rnf-like complex [20]. However, neither the genes for Ech nor the Rnf-like complex were found in the three *Methanosaeta* genomes; instead, a gene cluster comprised of 11 genes (Mhar_1410-Mhar_1420) for a F_420_H_2_ dehydrogenase (Fpo) complex was present in the three genomes.

The F_420_H_2_ dehydrogenase complex (Fpo) functions specifically in methylotrophic methanogenesis in *Methanosarcina* spp. and the obligate methylotrophic methanogens, like *Methanohalophilus mahii* [21]. However, no gene for the FpoF subunit was found in the three genomes. This cytoplasmic protein accepts reducing equivalents from F_420_H_2_. Welte and Deppenmeier (2011) determined that *M. thermophila* uses reduced ferredoxin to reduce coenzyme M-coenzyme B heterodisulfide and proposed that *M. thermophila* uses the Fd: heterodisulfide oxidoreductase encoded by an Fpo operon as the energy-conserving system [22]. The activity of Fd: heterodisulfide oxidoreductase was also detected in the membrane fraction of an *Msr. mazei* ***Δ*** *ech* mutant [23], implying that other protein complexes, like the Fpo complex, may complete the activity in place of Ech.

## Materials and Methods

### DNA extraction

*Methanosaeta harundinacea* 6Ac^T^ ( = JCM 13211, CGMCC 1.5026, and DSM 17206) was isolated from a UASB reactor in our lab, the strain information and growth conditions were described previously [7]. Cells were harvested by centrifugation at 15,000×g for 15 min at 4°C from 1000 ml culture. The cell pellets were frozen at −80°C. Then the frozen cell pellets were placed into a sterile, pre-cooled mortar and put into liquid N_2_. After the liquid N_2_ had evaporated, the cells were ground to a powder with a rod. Upon being ground 5 times, the genomic DNA was extracted using the TIANamp Bacteria DNA Kit (TIANGEN Biotech, Beijing, China). The genomic DNA was quantified on 0.8% agarose gel stained with ethidium bromide and spectrophotometrically assessed.

### Genome sequencing, assembly, and gap closure

The genome sequence of *Methanosaeta harundinacea* 6Ac^T^ was determined using the Roche GS 454 system [24]. 220,740 reads containing up to 52,294,588 bases (averaged read length as 236 bp), were obtained resulting in a 26-fold coverage of the genome. Assembly was performed using the GS *de novo* Assembler software (http://www.454.com/) producing 62 contigs ranging from 500 bp to 160,686 bp (the N50 contig size is 92,019 bp). The relationship of the contigs was determined by multiplex PCR [25]. Gaps were then closed by sequencing the PCR products using ABI 3730xl capillary sequencers. Phred, Phrap, and Consed software packages (http://www.phrap.org/phredphrapconsed.html) were used for the final assembly and editing. Low quality regions of the genome were resequenced.

### Genome analysis and annotation

Putative CDSs were identified by GeneMark [26] and Glimmer [27]. Peptides shorter than 30 aa were eliminated. Sequences from the intergenic regions were compared to GenBank's non-redundant (nr) protein database [28] to detect pseudogenes and to identify genes missed by the Glimmer or GeneMark prediction. Insert sequences were first detected using the IS Finder database (http://www-is.biotoul.fr/is.html) with default parameters selected manually. Transfer RNA genes were predicted by tRNAScan-SE [29], while ribosomal DNAs (rDNAs) and other RNA genes were identified by comparing the genome sequence to the rRNA database [30] using the Infernal program [31]. Functional annotation of CDSs was performed through searching against the nr protein database using BLASTP [32]. The protein set was also searched against the COG (http://www.ncbi.nlm.nih.gov/COG/) [33] and KEGG (Kyoto encyclopedia of genes and genomes; http://www.genome.jp/kegg/) [34] databases for further function assignment. The criteria used to assign function to a CDS were (1) a minimum cutoff of 40% identity and 60% coverage of the protein length and (2) at least two best hits among the COG, KEGG, or nr protein database. A search for gene families in the genome was performed by BLASTCLUST. Subcellular localization of the proteins was predicted by the PSORTb program (v2.0.1) [35]. The TatP 1.0 server (v2.0) [36] and TATFIND 1.2 program [37] were used to detect the potential substrates of the Tat secretion system.

### Data availability

The sequence and annotation of the *M. harundinacea* chromosome and the plasmid were submitted to the GenBank database under accession numbers CP003117 and CP003118, respectively.

### RNA isolation and qRT-PCR

*M. harundinacea* 6Ac was grown in conditions described previously [8]. The cells were harvested for RNA isolation during the logarithmic growth phase at an OD_600 nm_ of 0.35–0.45. Total RNA was extracted using TRIzol® Reagent (Invitrogen) according to the manufacturer's instructions, modifications of the method included grinding in liquid nitrogen before TRIzol reagent was added. RNA was further purified by following the RNA cleanup protocol of the RNeasy Mini Kit (Qiagen). Contaminating DNA was digested twice with 0.1–0.2 U·µl^−1^ DNase I (Promega), according to the manufacturer's instructions, including 0.4–0.8 U·µl^−1^ RNasin (Promega) in the reaction. RNA was purified for a second time using the RNeasy Mini Kit prior to cDNA synthesis. 210–350 ng RNA and 500 ng random hexamers (Promega) were incubated for 10 min at 70°C, and subsequently cooled on ice. Synthesis of cDNA was performed in 1 mM of each dNTP (Promega), 16 U·µl^−1^ M-MLV reverse transcriptase (RNase H Minus, Point Mutation, Promega), 1.6 U·µl^−1^ RNasin Inhibitor (Promega) and one fold concentrated first strand buffer (Promega) for 10 min at room temperature, followed by 50 min at 45°C. Real-time PCR oligonucleotide primers (Table S1) were designed using the software Beacon Designer 5.0 (Premier Biosoft, Palo Alto, USA) to obtain maximal amplification efficiency and sensitivity. The specificity of primers was verified by evaluating the qPCR melting curve and PCR products by DNA gel electrophoresis. For quantification of gene expression, a DNA fragment including the target gene was amplified by PCR and quantified using a UV800 spectrophotometer (Beckman). These fragments were then serially diluted 10-fold (10^−3^–10^−9^) to be used for the standard curve, which was performed in triplicate.

Transcript quantification was performed using an ABI 7000 SDS® (Applied Biosystems™) with SYBR Green supermix (TAKARA, Dalian, China) using the following thermocycling program: 40 cycles of 95°C for 10 s and 60°C for 30 s. The copy number in each cDNA sample was calculated according to the calibration curve generated by the PCR products including the target gene.

### Western blot for the proteins participating in methyl-group oxidation pathway

For antibody production, FwdC, Mch, and McrD were heterologously produced in *E. coli* DH5α. The *fwdC* (Mhar_0373), *mch* (Mhar_2174), and *mcrD* (Mhar_0496) genes were cloned into pET-28a. PCR amplification was performed using *Pfu* DNA polymerase (Promega, Madison, USA). The *fwdC*, *mch*, and *mcrD* PCR fragments were generated with the primers 5′- GGGTGGTCCATATGAGGGAGATCA -3′ and 5′- TGTCAAAGCTTCAGGCCTCCAGGGA-3′; 5′-AAACATATGCTAGACTACGCCGAT-3′ and 5′- ATAAAGCTTAACCTTCACGGTTGG-3′; 5′-AATACATATGGTCACTAAATCA- GACACG-3′ and 5′- GAATTCATGTGTCACCGCCTG- 3′, respectively. The resulting construct was checked by sequencing (Biosune, Beijing, China). Then protein production was induced and purified as described previously [38]. Polyclonal antibodies against FwdC, McrD, and Mch were prepared at the Laboratory Animal Center, Institute of Genetics and Developmental Biology, Chinese Academy of Sciences, by injecting rats with the three purified recombinant proteins.

*M. harundinacea* 6Ac cells at different growth phases were harvested by centrifugation at 12,000×*g* and washed three times with PBS buffer. Cell suspensions were lysed by sonication and centrifuged at 21,000×*g* to obtain the cell fractions. Supernatant fractions were used as all protein of cell extract. The fractions were subjected to SDS-PAGE on a 12% gel and then transferred to nitrocellulose membranes (Amersham, Little Chalfont, UK). Western blot and immunodetection were performed as described previously [39]. The non-specific binding of antibodies was blocked by incubation with Tris-buffered saline and Tween-20 (TBST) with 5% skim milk. Membranes were then probed with a 1,000-fold dilution of the following polyclonal antibodies: rat anti-FwdC, rat anti-Mch, and rat anti-McrD. Bound antibodies were visualized using goat anti-rat IgG (Cwbio, China) conjugated to horseradish peroxidase (HRP), followed by enhanced chemiluminescence (Amersham) according to manufacturer's instructions

### Incorporation of ^13^C-labeled acetate, methane, and bicarbonate

*M. harundinacea* was cultured in basal medium omitting NaHCO_3_ containing 50 mM acetate by following the procedure previously described [8]. The ^13^C labeled acetate or ^13^C labeled bicarbonate was added to the culture at a final concentration of 5% (w/w) of non-labeled substrate. Cells were cultured in 100-ml serum vials containing 50 ml of medium (pH 7.0 at 25°C) under an atmosphere of N_2_ and incubated at 37°C. Methane and acetate were determined by gas chromatograph GC-14B (Shimadzu) and CO_2_ was determined by gas chromatograph GC-14C (Shimadzu) [8], [40]. The stable isotope composition was determined by Trace GC/IsoLink/Delta V Advantage GC/IRMS (Thermo Fisher Scientific, Bremen, Germany) [41], [42].

## Supporting Information

Table S1 **The primers used in this study.**
(DOC)

Table S2 **The accession numbers of the proteins included in** **Figure 3** **.**
(DOC)
