# Supplementary material for: The Genome Characteristics and Predicted Function of Methyl-Group Oxidation Pathway in the Obligate Aceticlastic Methanogens, Methanosaeta spp
Source: PLoS One. 2012 May 10;7(5):e36756. doi: 10.1371/journal.pone.0036756 (PMC3349665; doi:10.1371/journal.pone.0036756)
Supplement: Table S1 — The primers used in this study. (DOC) [file pone.0036756.s001.doc]

**Table S1. The primers used in this study**

| Gene | Sense Primer | Anti_sense Primer |
| --- | --- | --- |
| Mhar_0751 | CTCCTATGTCGCCTATGG | CTTCTGCTCCTGGATGAT |
| Mhar_0789 | CACCATCACAGGCTTCTA | GGAAGGATCACTACGAAGA |
| Mhar_2323 | CCTTCGGCTATCCTGTAG | AACTTGGTGCTCTTCAGA |
| Mhar_0495 | TCGTCACTCAGTTCAAGG | CTGCTCGTAGATGGAAGA |
| Mhar_0498 | ACAAGAAGTTCGGAAGGGACTTCG | GTTGTACATGGAGATGCCACGCTT |
| Mhar_2090 | GTCACCATCTCCTACATCA | CTCCCTCTCTCCTCCATA |
| Mhar_2091 | ACATAATTGAGATCGTCATTGG | ATGCTGTCGGTGTAGTTC |
| Mhar_2092 | ACAAGACGAAGCTGATGA | GCCAGGAACGAGAAGTAA |
| Mhar_2093 | GATGATCGCAGAGGAGAG | ATGAACCCGTACCAGATG |
| Mhar_2094 | CGATCAGGAAGGAAGGAT | TATCTGCTGCTTGAACCT |
| Mhar_2095 | GGCAGATGAAGAGATTGTG | CAAGAGGACCATCAGGAT |
| Mhar_2096 | CCAAGATCGACGAGAAGA | CCGCCAGCAAATAGTATG |
| Mhar_2097 | GATCCACAACTCCATCAAC | TTGAAGGCGAGGACTATG |
| Mhar_0792 | CACCATCACAGGCTTCTA | GGAAGGATCACTACGAAGA |
| Mhar_0793 | GTACAAGATCGCCAAGTTC | GTAGTAGGTATCTTCCGTGAA |
| Mhar_0604 | GCCTACGGACTTCAGATTA | CGAACTTCTTCTCCAGGA |
| Mhar_0605 | CCTCAAGACGATCTACGA | CAGCAGGAACTCCGATAT |
| Mhar_0607 | CCAGTATCCGACGAACAT | GCACCAGATGATCTTGAC |
| Mhar_0373 | GGTTTCTGGTCCCTGTAG | GGAGGAGGAGATAAGCATC |
| Mhar_0374 | CCTCCATACGAACAACCT | ACTGCTGGTGTCTATTGAA |
| Mhar_0375 | GACCTGATCGTCTTCTGG | GCCTTATGAGGATGAACTTATC |
| Mhar_0376 | CTGTGAGGCGAAGATGTC | GCCCATCGGTATGAAGAT |
| Mhar_1283 | AGATGGTGGACATGATGAA | GGTAGCCGTAGAGGTAAG |
| Mhar_1284 | GCCTACAAGATGGAATGC | GATAGACCCGTCCTCTCT |
| Mhar_1285 | GGCGAGATCGAGATAATGA | CATCCGTCACATCAATATCAA |
| Mhar_1286 | CGACTCCGATGAAGTGATA | AAGCCGATGGTGTAGTAC |
| Mhar_1287 | CTGGTGATAGGCTCTGTG | GCGAAGTTGAGGTAGGAA |
| Mhar_1288 | ATTGGAGGAGGTCATAAGAT | CCTTCGGGCTATGTCATA |
| Mhar_1285 | GGCGAGATCGAGATAATGA | CATCCGTCACATCAATATCAA |
| Mhar_1286 | CGACTCCGATGAAGTGATA | AAGCCGATGGTGTAGTAC |
| Mhar_1287 | CTGGTGATAGGCTCTGTG | GCGAAGTTGAGGTAGGAA |
| Mhar_1288 | ATTGGAGGAGGTCATAAGAT | CCTTCGGGCTATGTCATA |
| Mhar_2214 | TTCTGATCACGGCGATAACGGA | CCTCCAGGTTCTTGAAGCTCATGT |
| Mhar_2174 | CTTGGCGGGAGCGAAGAC | TACGGCGGCGAGACCTACT |
| Mhar_1470 | GGGCATCGGAGTTGAAGAGG | TCGGAGCCAAGATGACGGT |
| Mhar_0856 | CGCCACCGAATCCCTGAC | TCACCGATCACTACAACAACCG |
