# Supplementary material for: The Genome Characteristics and Predicted Function of Methyl-Group Oxidation Pathway in the Obligate Aceticlastic Methanogens, Methanosaeta spp
Source: PLoS One. 2012 May 10;7(5):e36756. doi: 10.1371/journal.pone.0036756 (PMC3349665; doi:10.1371/journal.pone.0036756)
Supplement: Table S2 — The accession numbers of the proteins included in Figure 3 . (DOC) [file pone.0036756.s002.doc]

**Table S2. The accession numbers of the proteins included in Figure 3**

| **Enzyme** | **Accession number** | **Organism** |
| --- | --- | --- |
| Ftr | Mhar_2214 | *Methanosaeta harundinacea* 6Ac |
| Ftr | YP_004383947 | *Methanosaeta concilii* GP6 |
| Ftr | YP_844050 | *Methanosaeta thermophila* PT |
| Ftr | NP_614984 | *Methanosarcina acetivorans* C2A |
| Ftr | YP_304532 | *Methanosarcina barkeri* Fusaro |
| Ftr | NP_633345 | *Methanosarcina mazei* Go1 |
| Ftr | YP_566370 | *Methanococcoides burtonii* DSM 6242 |
| Ftr | YP_003727460 | *Methanohalobium evestigatum* Z-7303 |
| Ftr | YP_003543165 | *Methanohalophilus mahii* DSM 5219 |
| Ftr | YP_004617074 | *Methanosalsum zhilinae* DSM 4017 |
| Ftr | YP_004521004 | *Methanobacterium sp.* SWAN-1 |
| Ftr | YP_003424764 | *Methanobrevibacter ruminantium* M1 |
| Ftr | YP_003246838 | *Methanocaldococcus vulcanius* M7 |
| Ftr | YP_003356088 | *Methanocella paludicola* SANAE |
| Ftr | YP_001323435 | *Methanococcus vannielii* SB |
| Ftr | YP_001029658 | *Methanocorpusculum labreanum* Z |
| Ftr | YP_001046543 | *Methanoculleus marisnigri* JR1 |
| Ftr | YP_003895664 | *Methanoplanus petrolearius* DSM 11571 |
| Ftr | YP_448514 | *Methanosphaera stadtmanae* DSM 3091 |
| Ftr | YP_002467260 | *Methanosphaerula palustris* E1-9c |
| Ftr | YP_503247 | *Methanospirillum hungatei* JF-1 |
| Ftr | YP_004575931 | *Methanothermococcus okinawensis* IH1 |
| Ftr | YP_004004097 | *Methanothermus fervidus* DSM 2088 |
| Mch | Mhar_2174 | *Methanosaeta harundinacea* 6Ac |
| Mch | YP_004383804 | *Methanosaeta concilii* GP6 |
| Mch | YP_842689 | *Methanosaeta thermophila* PT |
| Mch | NP_616637 | *Methanosarcina acetivorans* C2A |
| Mch | YP_305738 | *Methanosarcina barkeri* Fusaro |
| Mch | NP_634677 | *Methanosarcina mazei* Go1 |
| Mch | YP_565374 | *Methanococcoides burtonii* DSM 6242 |
| Mch | YP_003727251 | *Methanohalobium evestigatum* Z-7303 |
| Mch | YP_003542289 | *Methanohalophilus mahii* DSM 5219 |
| Mch | YP_004616412 | *Methanosalsum zhilinae* DSM 4017 |
| Mch | YP_004519292 | *Methanobacterium sp.* SWAN-1 |
| Mch | YP_003424361 | *Methanobrevibacter ruminantium* M1 |
| Mch | YP_003247696 | *Methanocaldococcus vulcanius* M7 |
| Mch | YP_003356187 | *Methanocella paludicola* SANAE |
| Mch | YP_001323026 | *Methanococcus vannielii* SB |
| Mch | YP_001029842 | *Methanocorpusculum labreanum* Z |
| Mch | YP_001046583 | *Methanoculleus marisnigri* JR1 |
| Mch | YP_003893284 | *Methanoplanus petrolearius* DSM 11571 |
| Mch | YP_448258 | *Methanosphaera stadtmanae* DSM 3091 |
| Mch | YP_002467046 | *Methanosphaerula palustris* E1-9c |
| Mch | YP_501925 | *Methanospirillum hungatei* JF-1 |
| Mch | YP_004577331 | *Methanothermococcus okinawensis* IH1 |
| Mch | YP_004003819 | *Methanothermus fervidus* DSM 2088 |
| Mer | Mhar_0856 | *Methanosaeta harundinacea* 6Ac |
| Mer | YP_004384673 | *Methanosaeta concilii* GP6 |
| Mer | YP_842643 | *Methanosaeta thermophila* PT |
| Mer | NP_618605 | *Methanosarcina acetivorans* C2A |
| Mer | YP_303818 | *Methanosarcina barkeri* Fusaro |
| Mer | NP_632652 | *Methanosarcina mazei* Go1 |
| Mer | YP_566974 | *Methanococcoides burtonii* DSM 6242 |
| Mer | YP_003727762 | *Methanohalobium evestigatum* Z-7303 |
| Mer | YP_003542654 | *Methanohalophilus mahii* DSM 5219 |
| Mer | YP_004615267 | *Methanosalsum zhilinae* DSM 4017 |
| Mer | YP_004520699 | *Methanobacterium sp.* SWAN-1 |
| Mer | YP_003423312 | *Methanobrevibacter ruminantium* M1 |
| Mer | YP_003246509 | *Methanocaldococcus vulcanius* M7 |
| Mer | YP_003357736 | *Methanocella paludicola* SANAE |
| Mer | YP_001323585 | *Methanococcus vannielii* SB |
| Mer | YP_001029521 | *Methanocorpusculum labreanum* Z |
| Mer | YP_001046472 | *Methanoculleus marisnigri* JR1 |
| Mer | YP_003895366 | *Methanoplanus petrolearius* DSM 11571 |
| Mer | YP_448153 | *Methanosphaera stadtmanae* DSM 3091 |
| Mer | YP_002465432 | *Methanosphaerula palustris* E1-9c |
| Mer | YP_503681 | *Methanospirillum hungatei* JF-1 |
| Mer | YP_004575971 | *Methanothermococcus okinawensis* IH1 |
| Mer | YP_004003839 | *Methanothermus fervidus* DSM 2088 |
| Mtd | Mhar_1470 | *Methanosaeta harundinacea* 6Ac |
| Mtd | YP_004383615 | *Methanosaeta concilii* GP6 |
| Mtd | YP_843238 | *Methanosaeta thermophila* PT |
| Mtd | NP_619291 | *Methanosarcina acetivorans* C2A |
| Mtd | YP_304643 | *Methanosarcina barkeri* Fusaro |
| Mtd | NP_619291 | *Methanosarcina mazei* Go1 |
| Mtd | YP_565622 | *Methanococcoides burtonii* DSM 6242 |
| Mtd | YP_003727361 | *Methanohalobium evestigatum* Z-7303 |
| Mtd | YP_003541849 | *Methanohalophilus mahii* DSM 5219 |
| Mtd | YP_004615335 | *Methanosalsum zhilinae* DSM 4017 |
| Mtd | YP_004518857 | *Methanobacterium sp.* SWAN-1 |
| Mtd | YP_003424884 | *Methanobrevibacter ruminantium* M1 |
| Mtd | YP_003246651 | *Methanocaldococcus vulcanius* M7 |
| Mtd | YP_003356368 | *Methanocella paludicola* SANAE |
| Mtd | YP_001323869 | *Methanococcus vannielii* SB |
| Mtd | YP_001029524 | *Methanocorpusculum labreanum* Z |
| Mtd | YP_001046477 | *Methanoculleus marisnigri* JR1 |
| Mtd | YP_003895371 | *Methanoplanus petrolearius* DSM 11571 |
| Mtd | YP_447224 | *Methanosphaera stadtmanae* DSM 3091 |
| Mtd | YP_002465434 | *Methanosphaerula palustris* E1-9c |
| Mtd | YP_503679 | *Methanospirillum hungatei* JF-1 |
| Mtd | YP_004576424 | *Methanothermococcus okinawensis* IH1 |
| Mtd | YP_004004372 | *Methanothermus fervidus* DSM 2088 |
| MtrA | YP_004383630 | *Methanosaeta concilii* GP6 |
| MtrA | Mhar_2094 | *Methanosaeta harundinacea* 6Ac |
| MtrA | YP_843796 | *Methanosaeta thermophila* PT |
| MtrA | NP_615245 | *Methanosarcina acetivorans* C2A |
| MtrA | YP_304801 | *Methanosarcina barkeri* Fusaro |
| MtrA | NP_633567 | *Methanosarcina mazei* Go1 |
| MtrA | YP_004520861 | *Methanobacterium sp.* SWAN-1 |
| MtrA | YP_003424661 | *Methanobrevibacter ruminantium* M1 |
| MtrA | YP_003246504 | *Methanocaldococcus vulcanius* M7 |
| MtrA | YP_003355598 | *Methanocella paludicola* SANAE |
| MtrA | YP_566178 | *Methanococcoides burtonii* DSM 6242 |
| MtrA | YP_001323394 | *Methanococcus vannielii* SB |
| MtrA | YP_001030985 | *Methanocorpusculum labreanum* Z |
| MtrA | YP_001046521 | *Methanoculleus marisnigri* JR1 |
| MtrA | YP_003727040 | *Methanohalobium evestigatum* Z-7303 |
| MtrA | YP_003541579 | *Methanohalophilus mahii* DSM 5219 |
| MtrA | YP_003895594 | *Methanoplanus petroleariu*s DSM 11571 |
| MtrA | YP_004615837 | *Methanosalsum zhilinae* DSM 4017 |
| MtrA | YP_447357 | M*ethanosphaera stadtmanae* DSM 3091 |
| MtrA | YP_002467322 | *Methanosphaerula palustris* E1-9c |
| MtrA | YP_503598 | *Methanospirillum hungatei* JF-1 |
| MtrA | YP_004576699 | *Methanothermococcus okinawensis* IH1 |
| MtrA | YP_004004340 | *Methanothermus fervidus* DSM 2088 |
| MtrH | YP_004383627 | *Methanosaeta concilii* GP6 |
| MtrH | Mhar_2097 | *Methanosaeta harundinacea* 6Ac |
| MtrH | YP_844061 | *Methanosaeta thermophila* PT |
| MtrH | NP_615242 | *Methanosarcina acetivorans* C2A |
| MtrH | YP_304798 | *Methanosarcina barkeri* Fusaro |
| MtrH | NP_633564 | *Methanosarcina mazei* Go1 |
| MtrH | YP_004520858 | *Methanobacterium sp.* SWAN-1 |
| MtrH | YP_003424658 | *Methanobrevibacter ruminantium* M1 |
| MtrH | YP_003246507 | *Methanocaldococcus vulcanius* M7 |
| MtrH | YP_003355601 | *Methanocella paludicola* SANAE |
| MtrH | YP_566857 | *Methanococcoides burtonii* DSM 6242 |
| MtrH | YP_001323397 | *Methanococcus vannielii* SB |
| MtrH | YP_001030984 | *Methanocorpusculum labreanum* Z |
| MtrH | YP_001046520 | *Methanoculleus marisnigri* JR1 |
| MtrH | YP_003727648 | *Methanohalobium evestigatum* Z-7303 |
| MtrH | YP_003541580 | *Methanohalophilus mahii* DSM 5219 |
| MtrH | YP_003895591 | *Methanoplanus petroleariu*s DSM 11571 |
| MtrH | YP_004616778 | *Methanosalsum zhilinae* DSM 4017 |
| MtrH | YP_447360 | M*ethanosphaera stadtmanae* DSM 3091 |
| MtrH | YP_002467325 | *Methanosphaerula palustris* E1-9c |
| MtrH | YP_503599 | *Methanospirillum hungatei* JF-1 |
| MtrH | YP_004576696 | *Methanothermococcus okinawensis* IH1 |
| MtrH | YP_004004337 | *Methanothermus fervidus* DSM 2088 |
